# Chemical characterization of *Brickellia cavanillesii* (Asteraceae) using gas chromatographic methods

**DOI:** 10.1002/fsn3.52

**Published:** 2013-12-27

**Authors:** Etetor R Eshiet, Jinqiu Zhu, Todd A Anderson, Ernest E Smith

**Affiliations:** The Institute of Environmental and Human Health, Texas Tech UniversityLubbock, Texas, 79409-1163

**Keywords:** *Brickellia cavanillesii*, chromatography, lyophilize, spectrometry

## Abstract

A methanol extract of lyophilized *Brickellia cavanillesii* was quantitatively analyzed using gas chromatographic (GC) techniques. The chromatographic methods employed were (i) GC-flame ionization detector (GC-FID), (ii) GC-mass spectrometry (GC-MS), and (iii) purge and trap GC-MS (P&T GC-MS). Thirteen compounds were identified with a quality match of 90% and above using GC-MS. The compounds were (**1**) Cyclohexene, 6-ethenyl-6-methyl-1-(1-methylethyl)-3-(1-methylethylidene)-, (S)-; (**2**) Bicylo (2.2.1) heptan-2-one, 1, 7, 7-trimethyl-(1S, 4S)-; (**3**) Phenol, 2-methoxy-4-(1-propenyl)-; (**4**) Benzene, 1-(1, 5-dimethyl-4-hexenyl)-4-methyl-; (**5**) Naphthalene, 1, 2, 3, 5, 6, 8a-hexahydro4, 7-dimethyl-1-1-(1-methylethyl)-, (1S-cis)-; (**6**) Phenol, 2-methoxy-; (**7**) Benzaldehyde, 3-hydroxy-4-methoxy-; (**8**) 11, 13-Eicosadienoic acid, methyl ester; (**9**) 2-Furancarboxaldehyde, 5-methyl-; (**10**) Maltol; (**11**) Phenol; (**12**) Hydroquinone; (**13**) 1H-Indene, 1-ethylideneoctahydro-7a-methyl-, (1E, 3a.alpha, 7a.beta.). Other compounds (**14**) 3-methyl butanal; (**15**) (D)-Limonene; (**16**) 1-methyl-4-(1-methyl ethyl) benzene; (**17**) Butanoic acid methyl ester; (**18**) 2-methyl propanal; (**19**) 2-butanone; (**20**) 2-pentanone; and (**21**) 2-methyl butane were also identified when P&T GC-MS was performed. Of the 21 compounds identified, 12 were validated using chemical standards. The identified compounds were found to be terpenes, derivatives of terpenes, esters, ketones, aldehydes, and phenol-derived aromatic compounds; these are the primary constituents of the essential oils of many plants and flowers.

## Practical Application

*Brickellia cavanillesii* (Asteraceae), one of the popular herbal plants consumed in Central America, Mexico, and the southwestern parts of the U.S.A. for the treatment of Type 2 diabetes mellitus, is believed to possess hypoglycemic and antioxidative properties. Unfortunately, little is known about its chemical composition. This study investigates the lyophilized extracts of *B. cavanillesii* in an attempt to elucidate its use as a therapeutic agent.

## Introduction

The use of complementary and alternative medicine (CAM) in disease therapy has increased tremendously in recent years. Statistical data indicate that diabetes patients are 1.6 times more likely to use CAM treatment than people without diabetes and invariably are more susceptible to the attendant consequences of improper CAM usage (Egede et al. 2002; Shane-McWhorter 2007). The dearth of valid ethno botanical information on herbal plants used in diabetes therapy remains an impediment to the utilization of CAM by clinicians. Factors such as improper identification and standardization of these herbal plants constitute a potential reason for concern. One such herbal plant used in the treatment of diabetes is *Brickellia*. *Brickellia* is a member of the Asteraceae botanical family universally known as brickellbushes. It consists of about 380 genera (∼3000 species) and is among the more basal lineages of the Eupatorieae. *Brickellia* species are native to Mexico and the southwestern portion of the United States; they are usually perennial shrubs that have creamy florets followed by one-seeded fruits in a prominent bristly sheath (Scott 1997; Schmidt and Schilling 2000). As an intervention, *Brickellia* is traditionally consumed as an herbal tea for its presumed antiulcer, antimigraine, cardiotonic, and antidiabetic properties among others. The extant literature reports that the chemical components of certain *Brickellia* species have hypoglycemic and antioxidative properties (Marles and Farnsworth 1995; Andrade-Cetto and Heinrich 2005; Rivero-Cruz et al. 2006). Studies conducted on the chemical characterization of *Brickellia veronicaefolia* (Rivero-Cruz et al. 2006) have shown that 86 percent of its essential oil consists of benzoates and sesquiterpenoids. Another study indicates that one flavone, 5, 7, 3′-trihydroxy-3, 6, 4′-trimethoxyflavone, has been isolated from the leaves of *Brickellia veronicaefolia* Gray (Perez et al. 2002). In a study performed on *Brickellia cavanillesii,* a natural product, 6-acetyl-5-hydroxy-2, 2-dimethyl-2H-chromene (C_13_H_14_O_3_), was isolated (Rodriguez-Lopez et al. 2006).

It has been acknowledged that the enormous cost of modern treatment has led many people in underdeveloped and developing countries to use traditional or alternative medicine. Herbal plants are a primary source of alternative medicine. A scientific investigation of traditional herbal plants may provide valuable leads for the development of alternative drugs and therapeutic remedies. Treatment using herbal extracts constitutes a tremendous resource for alternative remedy especially at disease onset. Presently, considerable lack of knowledge continues to exist with regards to the therapeutic and pharmacodynamic potential of the individual chemicals of herbal plants. This has made it difficult to determine the efficacy of most herbal plants used in therapy. It is therefore essential that scientific evidence be established regarding the efficacy and pharmacodynamic potential of the individual bioactive chemicals of herbal supplements. Accurate identification and determination of the chemical components of herbal plants will aid in predicting their biological actions. This will lead to the development of reliable and reproducible pharmacological and clinical assays.

Our plant of interest is *B. cavanillesii* known in many Latin American countries as “prodigiosa” or “hamula.” It is available commercially in herbal stores and is presently and commonly used by diabetics as a cheaper alternative to insulin. For this study we chemically characterized the individual chemical components of *B. cavanillesii* using gas chromatographic methods. Identified compounds were validated by comparing the retention time and mass spectra of samples with standards. Gas chromatography with flame ionization detection (GC-FID) was used for method development. Subsequently GC coupled with mass spectrometry (GC-MS) analysis was used for confirmation. Purge and trap GC MS (P&T GC-MS) analysis was used for the elucidation of the more volatile compounds present in *Brickellia* extracts. Customarily, *B. cavanillesii* is prepared for consumption by boiling the plant (aerial) in water (Poss et al. 2003). Samples prepared for analysis were prepared similarly and subsequently lyophilized. The lyophilization technique was adopted because it ensures standardized composition of the herbal extracts (fixed dose), and resistance to decomposition by heat and humidity. The aim of this study was determination of the chemical composition of the tea prepared from lyophilized *B. cavanillesii*.

## Material and Methods

### General experimental procedure

Chromatographic measurements of tea sample: GC-FID measurements were conducted on a Hewlett-Packard (Palo Alto, CA) 6890 GC with a FID. The GC column (75 m × 530 μm diameter) was a DB-624 capillary column (J&W Scientific, Folsom, CA). The temperature program was linear with a 5 min hold at 35°C, ramping to 260°C at a rate of 5°C/min and holding for 4 min, for a total run time of 54 min. The carrier gas used was He (47 mL/min). Injection volume was 3 μL using the split less mode. For the GC-MS analyses we employed identical chemistry as outlined for GC-FID. The GC-MS measurements were conducted on a Hewlett Packard 6890 series GC system with a HP 5973 Mass Selective Detector. The mass spectrometer was operated in the full scan mode with electron impact (EI+) ionization of 70 eV. The temperature of electron ionization source and MS quadrupole were kept at 230 and 150°C, respectively. For qualitative analysis, the MS detector was operated in full-scan mode from *m/z* 50 to 550 at 2.92 scans/sec. Control of the equipment, data acquisition, processing, and management of chromatographic information were performed by the Enhanced MSD D.02.00.275 software program. Constituents of *B. cavanillesii* were identified by matching their 70 eV mass spectra with those of the reference library. For P&T GC-MS analysis, an aliquot of 0.5 mL of tea extract in methanol was dissolved in 2.5 mL of Milli-Q (Billerica, MA) water. Measurements were conducted on an Agilent 6890 series GC system with an Agilent 5973 Mass Selective Detector (Santa Clara, CA), OI Analytical 4560 Sample Concentrator (College Station, TX) (purge pressure – 25 psi [170 kpa]) and OI Analytical DPM-16 Discrete Purging Multisampler. The GC column was a capillary column with model number HP 1909IV-402 (HP-624) (25 m × 200 μm diameter). The linear temperature range was from 35 to 240°C, at a rate of 12°C/min. The carrier gas used was He (17 mL/min).

#### Plant material

Leaves and twigs of a specimen of *B. cavanillesii* were purchased from an herbal store “Hieberia Claria” in Piedras Negras, Coahuila, Mexico.

#### Extraction and isolation

The leaves and twigs of *B. cavanillesii* were pulverized to a fine powder using the Black and Decker Coffee Bean Grinder (Towson, MA) “Smart Grind TM” C3G100W model. Five grams of *B. cavanillesii* was dissolved in 237 mL (+237 mg [0.1% ascorbic acid]) of hot (95°C) Milli-Q water (18.3 MΩ, pH 6.8). The ascorbic acid was added to prevent oxidation. The solution was thoroughly mixed and allowed to steep for 3 min. The filtrate was immediately frozen by immersion in liquid nitrogen and subsequently lyophilized using LABCONCO Freezone^1^ lyophilizer (Kansas City, MO). Five grams of *B. cavanillesii* yielded ∼1 g of lyophilized tea sample on average. Extraction was done by introducing 2 g of the lyophilized *B. cavanillesii* into 100% high-performance liquid chromatography (HPLC) grade methanol using Dionex ASE 200 Accelerated Solvent Extractor (Salt Lake, UT). Each extraction cycle included a 5 min preheating (100°C) step, immediately followed by a 5 min static extraction process with 100% HPLC grade methanol at constant temperature (100°C) and pressure (1500 psi). The resulting extract (15–20 mL/sample) was transferred and concentrated to 1 mL using nitrogen evaporation. The samples were subsequently filtered (0.45 μm; Acrodisc, Port Washington, NY) into autosampler vials.

### Preparation of chemicals used as standards for GC-MS study

Four chemicals were used as internal standards for GC-MS study. Standards were purchased commercially from Sigma-Aldrich (St Louis, MO) and were chosen because of their availability. The compounds chosen were (**2**) Bicyclo (2.2.1) heptan-2-one, 1, 7, 7-trimethyl-, (1S, 4S)-; (**6**) Phenol, 2-methoxy; (**9**) 2-Furancarboxaldehyde, 5-methyl-; and (**12**) Hydroquinone. An aliquot of 2 mL of a 0.1 molar solution in methanol (99% purity; density assumed to be 1.00 g/cm^3^) of the four respective chemical standards were prepared. Filtration was done using a 20 mL syringe with a 0.2 μm filter and the extract inserted into 2 mL GC/LC autosampler vials.

### Preparation of chemicals used as standards for P&T GC-MS study

Eight chemicals were used as standards for P&T GC-MS study. Standards were purchased from VWR (Radnor, PA), TCI America (Portland, OR), and Alfa Aesar (Ward Hill, MA). The compounds were (**14**) 3-methyl butanal; (**15**) (D)-Limonene; (**16**) 1-methyl-4-(-1-methyl ethyl) benzene; (**17**) Butanoic acid methyl ester; (**18**) 2-methyl propanal; (**19**) 2-butanone; (**20**) 2-pentanone; (**21**) 2-methyl butanal. One-half mL of 0.1 molar solution in methanol (99% purity; density assumed to be 1.00 g/cm^3^) of the eight respective chemical standards was dissolved in 2.5 mL of Milli-Q water. Filtration was done using a 20 mL syringe with a 0.2 μm filter and the extract inserted into 60 mL borosilicate glass vials. Measurements were conducted on an Agilent 6890 series GC system with an Agilent 5973 Mass Selective Detector, OI Analytical 4560 Sample Concentrator (purge pressure – 25 psi [170 kpa]) and OI Analytical DPM-16 Discrete Purging Multisampler. The GC column was a capillary column with model number HP 1909IV-402 (HP-624) (25 m × 200 μm). The linear temperature range was from 35 to 240°C, at a rate of 12 mL/min. The carrier gas used was He (17 mL/min).

## Results and Discussion

**Figure 1:**
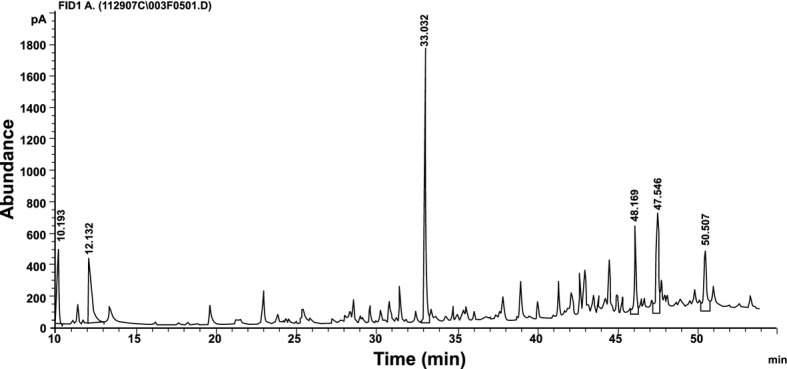
Gas chromatography-flame ionization detector (GC-FID) profile of the methanolic extract of lyophilized *Brickellia cavanillesii*.

GC-FID was used to develop a method that ensured high separation efficiency. The chromatogram obtained using this method exhibited good separation as illustrated in Figure 1.

Consequently this method was used for GC-MS. A total of 109 peaks were obtained using GC-MS 70 eV mass spectra.

**Figure 2:**
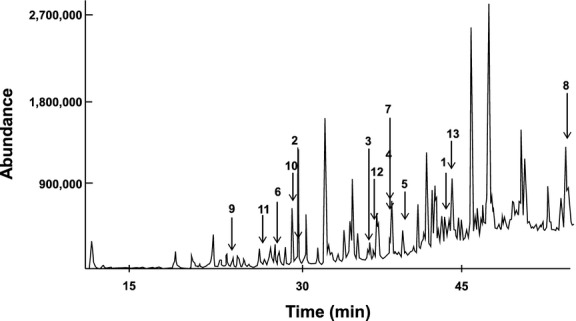
Gas chromatography-mass spectrometry (GC-MS) profile of the methanolic extract of lyophilized *Brickellia cavanillesii* showing compounds with a quality match of 90% and above. 1, Cyclohexene, 6-ethenyl-6-methyl-1-(1-methylethyl)-3-(1-methylethylidene)-, (S)-; 2, Bicylo (2.2.1) heptan-2-one, 1, 7, 7-trimethyl-(1S, 4S)-; 3, Phenol, 2-methoxy-4-(1-propenyl)-; 4, Benzene, 1-(1, 5-dimethyl-4-hexenyl)-4-methyl-; 5, Naphthalene, 1, 2, 3 5, 6, 8a-hexahydro-4, 7-dimethyl-1-1-(1-methylethyl)-, (1S-cis)-; 6, Phenol, 2-methoxy-; 7, Benzaldehyde, 3-hydroxy-4-methoxy-; 8, 11, 13-Eicosadienoic acid, methyl ester; 9, 2-Furancarboxaldehyde, 5-methyl-; 10, Maltol; 11, Phenol; 12, Hydroquinone; 13, 1H-Indene, 1-ethylideneoctahydro-7a-methyl-, (1E, 3a.alpha, 7a.beta.).

**Figure 3:**
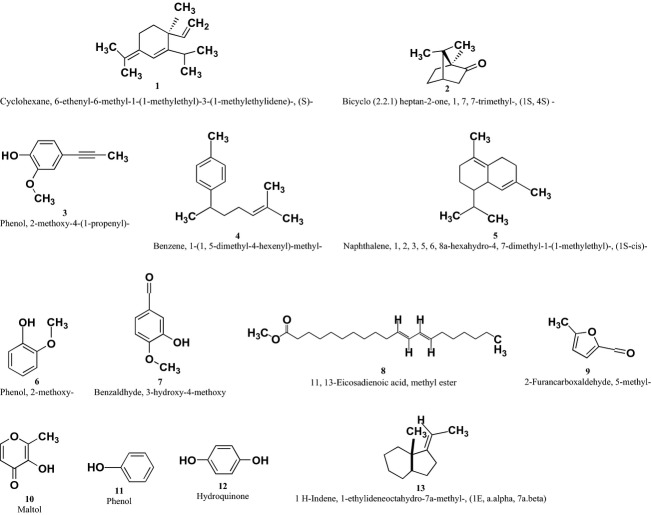
Chemical structures of the compounds (1–13) found in the methanolic extract of *Brickellia cavanillesii* identified by gas chromatography-mass spectrometry (GC-MS).

Thirteen of the compounds identified using GC-MS (see Fig. 2) had a quality match of 90% and above (Table 1). The compounds identified were **1**–**13** (Fig. 3).

**Table 1:** Gas chromatography-mass spectroscopy (GC-MS) results of the bioactive compounds identified in the methanolic extract of *Brickellia cavanillesii* listed by quality match

Chemical compounds identified by GC-MS		Quality match (%)	Retention time (*t*_R_) min
1	Cyclohexene, 6-ethenyl-6-methyl-1-(1-methylethyl)-3-(1-methylethylidene)-, (S)-;	97	42.5
2	Bicyclo (2.2.1) heptan-2-one, 1, 7, 7-trimethyl-, (1S, 4S)-	96	29.3
3	Phenol, 2-methoxy-4-(1-propenyl)-	95	35.6
4	Benzene, 1-(1, 5-dimethyl-4-hexenyl)-4-methyl-	95	37.6
5	Naphthalene, 1, 2, 3, 5, 6, 8a-hecahydro-4, 7-dimethyl-1-(1-methylethyl)-, (1S-cis)-	94	38.7
6	Phenol, 2-methoxy	94	27.6
7	Benzaldehyde, 3-hydroxy-4-methoxy-	94	37.9
8	11, 13 Eicosadienoic acid, methyl ester	94	53.3
9	2-Furancarboxaldehyde, 5-methyl-	91	23.4
10	Maltol	91	28.8
11	Phenol	90	25.8
12	Hydroquinone	90	36.3
13	1H-Indene, 1-ethylideneoctahydro-7a-methyl-, (1E, 3a.alpha, 7a.beta.)	90	43.1

**Figure 4:**
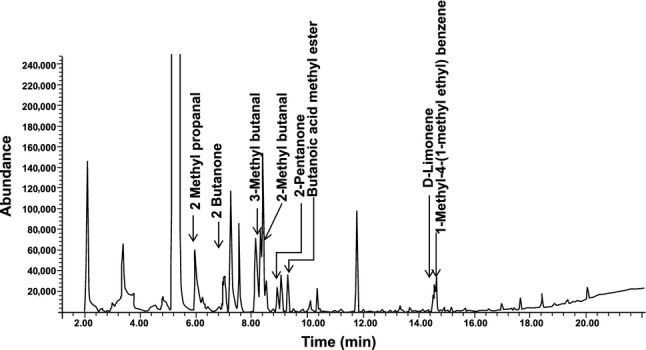
Purge and trap gas chromatography-mass spectrometry (P&T GC-MS) profile of the methanolic extract of lyophilized *Brickellia cavanillesii*.

**Figure 5:**
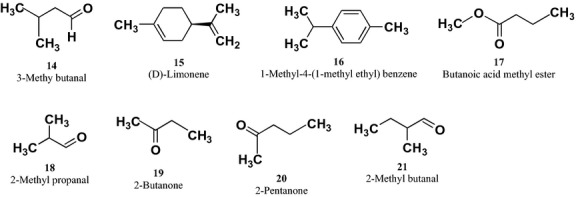
Chemical structure of the compounds (14–21) found in the methanolic extract of *Brickellia cavanillesii* identified by purge and trap gas chromatography-mass spectrometry (P&T GC-MS).

Eight compounds were identified using P&T GC-MS (Fig. 4; Table 2). These compounds were **14**–**21** (Fig. 5).

**Table 2:** Purge and trap gas chromatography-mass spectroscopy (P&T GC-MS) results for bioactive compounds identified in the methanolic extract of lyophilized *Brickellia cavanillesii* listed by quality match

Chemical compounds identified by P&T GC-MS		Quality match (%)	Retention time (*t*_R_) min
14	3-Methyl butanal	97	8.1
15	(D)-Limonene	94	14.4
16	1-Methyl-4-(1-methylethyl) benzene	94	14.5
17	Butanoic acid methyl ester	91	9.2
18	2-Methyl propanal	91	9.2
19	2-Butanone	90	6.9
20	2-Pentanone	78	8.9
21	2-Methyl butanal	50	8.3

**Figure 6:**
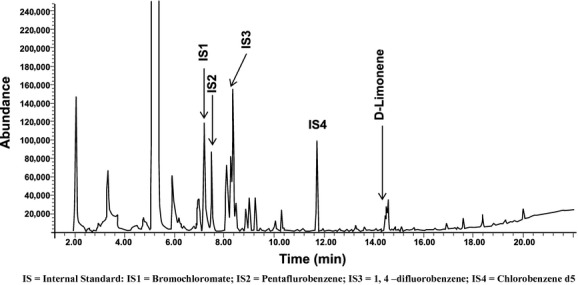
Purge and trap gas chromatography-mass spectroscopy (P&T GC-MS) profile of the methanolic extract of lyophilized *Brickellia cavanillesii* indicating retention time of (D)-Limonene.

**Figure 7:**
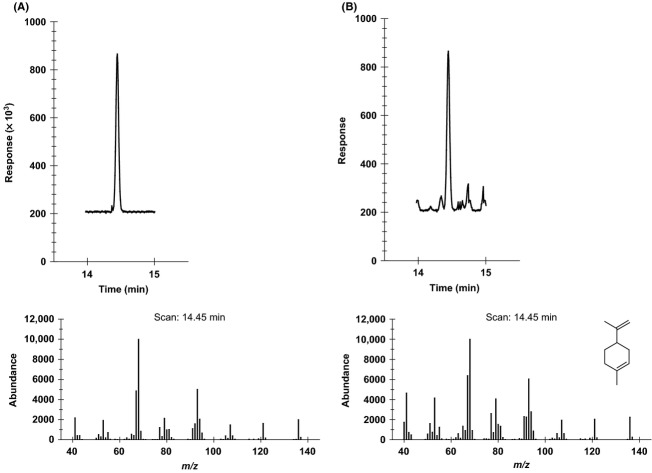
(A) Purge and trap gas chromatography-mass spectroscopy (P&T GC-MS) profile of (D)-Limonene standard. (B) P&T GC-MS profile of (D)-Limonene in a sample.

We validated 12 of the 21 identified compounds using reference standards purchased commercially from VWR, TCI America, and Alfa Aesar. Nine of the 21 compounds that were identified were not available for chemical confirmation. Validation of these compounds was performed by comparing the retention times of the isolated compounds against that of their reference standards. The retention times of the identified compounds were found to match that of the reference standards thus providing a reasonable confirmation of their identity. The validated compounds were **2**,**6**,**9**,**12**,**14**–**21**. An example using one of the validated compounds, **15** (D-Limonene), is illustrated using Figures 6, 7A, and 7B.

**Figure 8:**
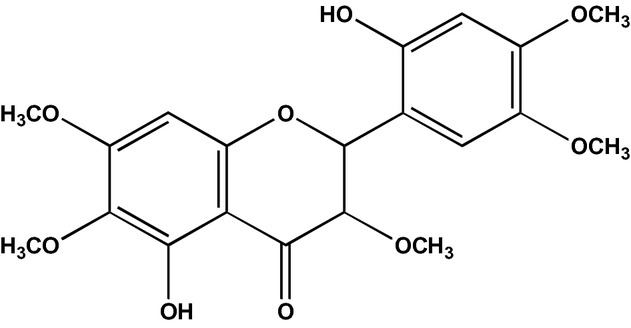
Brickellin.

There is very limited empirical and pharmacological evidence to validate the chemical and biological properties of *B. cavanillesii*. Extant data obtained from a docket submitted to the Department of Health and Human Services (HHS), Public Health Service, Food and Drug Administration (FDA), on 15 April 1997 states that the chemical composition of *B. cavanillesii* plants consists of a glycoside named Brickellin, resin, essential oil, fat, tannin, coloring material, gum, starch, chlorophyll, and mineral salts. Brickellin and resin were indicated as the main substances. Brickellin, 2′,5-dihydroxy-3, 4′, 5′, 6, 7-pentamethoxyflavone (Inhuma et al. 1985) (Fig. 8), has the molecular formula C_20_H_20_O_9_. The structure was determined using proton nuclear magnetic resonance (^1^HNMR), carbon-13 NMR (^13^CNMR), and electron impact mass (EIM) spectra.

Although literature suggests that Brickellin is a major component of *B. cavanillesii*, it does not state its relative content. Brickellin is an *O*-methylated flavonol. In vitro investigations suggest that flavonoids possess antiinflammatory, antimicrobial, antiallergic, anticancer, and cardiovascular properties (Yamamoto and Gaynor 2001; Cushnine and Lamb 2005; Ruela de Sousa et al. 2007; Tapas et al. 2008). Flavonoids also have significant antioxidant and antidiabetic activity (Fawzy et al. 2008). O-methylated flavonoids for the most part demonstrate superior pharmacological properties than their corresponding hydroxylated derivatives (Bernini et al. 2011). Consequently, we postulate that Brickellin may account, to some extent, for the supposed antidiabetic properties of *B. cavanillesii*. However, further studies need to be undertaken to establish the relative content of Brickellin in *B. cavanillesii* and also explore its potential antidiabetic properties. Most of the compounds positively identified using standards were found to have a relative content below 1.5% of the total content of lyophilized *B. cavanillesii*. These compounds **2**,**6**,**9**,**12**,**14**–**21** are terpenes, derivatives of terpenes, esters, ketones, aldehydes, and phenol-derived aromatic compounds. These are the primary constituents of the essential oils of many plants and flowers. Essential oils are used extensively in traditional medicine as antiseptic, antimicrobial, virucidal, fungicidal, analgesic, sedative, antiinflammatory, spasmolytic, and anesthesia. They are also used as fragrances in perfumery and also as preservatives and natural flavors for food (Sabu and Kuttan 2002; Burt 2004; Zareba et al. 2005; Bakkali et al. 2008). Essential oils are also known to have antioxidative properties (Yamamoto and Gaynor 2001; Cushnine and Lamb 2005; Ruela de Sousa et al. 2007; Tapas et al. 2008). Bicyclo (2.2.1) heptan-2-one, 1, 7, 7-trimethyl-, (1S, 4S) – otherwise known as camphor–is used in several cough preparations as a suppressant and topical analgesic. Camphor is also commonly used externally to relieve arthritis and rheumatic pains, neuralgia, and back pain. Camphor may be administered orally in small quantities (50 mg) for minor heart symptoms and fatigue. Conversely, in large doses, camphor is poisonous when ingested and can cause seizure, confusion, irritability, neuromuscular hyperactivity, and hepatotoxicity (Uc et al. 2000; Martin et al. 2004). Although data in literature suggest that the compounds we positively identified have medicinal properties, there is no evidence to show that they account for the presumed antidiabetic properties of *B. cavanillesii*. Presently investigations are being conducted at our laboratory at The Institute of Environmental and Human Health (TIEHH), Texas Tech University, to explore the therapeutic or ethnotoxicological potential of *B. cavanillesii* in the therapy of diabetes mellitus. It is hoped that results generated will elucidate the use of *B. cavanillesii* as a complementary and alternative agent in the therapy of Type 2 diabetes.
